# Feasibility of integrating genetic risk and digital health tools for cardiovascular prevention: the FitPreV protocol

**DOI:** 10.3389/fpubh.2026.1800961

**Published:** 2026-05-29

**Authors:** Diego Maria Tona, Martina Porcelli, Matteo Di Pumpo, Felicia Cuoco, Michela Francesca Cuoco, Leonardo Terenzi, Luciano Antonaci, Enrica Maggiori, Roberto Colletti, Emmanuela Guiducci, Massimo Mammucari, Filippo Paoletti, Giulio Pasca, Giulia Antonia Marra, Daniela Carbone, Tina Pasciuto, Tommaso Sanna, Giovanna Liuzzo, Edoardo Franceschini, Anna Villarini, Giancarlo Pocetta, Chiara de Waure, Stefania Boccia, Roberta Pastorino

**Affiliations:** 1 Section of Hygiene, University Department of Life Sciences and Public Health, Università Cattolica del Sacro Cuore, Rome, Italy; 2 General Practitioner, ASL RM 1, Rome, Italy; 3 Research Core Facility Data Collection G-STeP, Fondazione Policlinico Universitario A. Gemelli IRCCS, Rome, Italy; 4 Department of Cardiovascular and Pulmonary Sciences, Università Cattolica School of Medicine, Rome, Italy; 5 Department of Cardiovascular Science, Fondazione Policlinico Gemelli - IRCCS, Rome, Italy; 6 Department of Medicine and Surgery, University of Perugia, Perugia, Italy; 7 Department of Woman and Child Health and Public Health, Fondazione Policlinico Universitario A. Gemelli IRCCS, Rome, Italy

**Keywords:** cardiovascular diseases, digital health, genetic testing, primary prevention, public health, polygenic risk scores, wearable health devices

## Abstract

**Background:**

Cardiovascular diseases (CVDs) remain the leading cause of morbidity and mortality worldwide. While a substantial proportion of CVD burden is attributable to modifiable risk factors, individual genetic susceptibility also contributes significantly. Polygenic risk scores (PRS) have emerged as promising tools to quantify inherited cardiovascular risk, while digital health technologies, such as wearable health devices (WHDs), provide new opportunities for continuous lifestyle monitoring However, the feasibility of integrating PRS and WHDs into primary prevention within public healthcare systems has not yet been systematically evaluated.

**Objective:**

The FitPreV study aims to assess the feasibility of implementing PRS and WHDs in cardiovascular primary prevention among individuals with metabolic syndrome in the Italian National Health Service (SSN), focusing on their acceptability, practicality, and barriers to adoption in general practice.

**Methods:**

FitPreV is a pilot, four-arm, randomized controlled feasibility study involving 120 adults aged 40–69 years diagnosed with metabolic syndrome and an estimated 10-year CVD risk between 2.5 and 10%. Participants will be randomly assigned to: (1) standard counseling; (2) counseling plus WHD; (3) counseling plus PRS; or (4) counseling plus both PRS and WHD. Data will be collected at baseline, 1, 6, and 12 months. Feasibility indicators include recruitment and retention rates, acceptability, burden and structural readiness. Secondary outcomes include changes in lifestyle behaviors, lipid profiles and cardiovascular risk (SCORE2 algorithm).

**Expected results:**

The study will generate evidence on the operational feasibility and acceptability of integrating genetic risk assessment and digital monitoring tools into primary cardiovascular prevention within general practice settings.

**Conclusions:**

By evaluating real-world implementation challenges, FitPreV will provide foundational evidence to guide larger-scale effectiveness trials studying personalized prevention approaches into national primary care settings.

**Trial registration:**

https://clinicaltrials.gov/study/NCT06911294, identifier: NCT06911294.

## Introduction

Cardiovascular diseases (CVDs) encompass a wide array of conditions, including coronary artery disease, heart failure, and stroke, which arise from the complex interplay of numerous genetic variants, unhealthy lifestyles and diet (1) and socioeconomic factors (2). CVDs pose a significant global health concern (3), as they are the leading cause of death and illness both in Europe and worldwide (4). Significant progress in CVDs management, coupled with heightened awareness of the value of preventive strategies, has contributed to a global decline in age-standardized incidence and mortality rates. Nevertheless, as the population ages and grows, the absolute number of CVDs incident cases and deaths has increased over time (5). A considerable portion of the burden of CVDs can still be attributed to modifiable risk factors, including elevated systolic blood pressure, high levels of LDL cholesterol, and tobacco use (6). This highlights the importance of sustained efforts in counteracting CVDs and in the development of innovative strategies for prevention (7). In this context, personalization represents a cutting-edge area of medical research, enhancing population health while potentially lowering expenses for healthcare systems. Polygenic risk scores (PRS) have recently attracted attention for their ability to quantify an individual's genetic predisposition to CVDs (8). By identifying subjects with high inherited risk, PRS could enable earlier and more targeted preventive interventions, complementing traditional risk stratification tools (8). This is particularly relevant for individuals with metabolic syndrome, a cluster of conditions including central obesity, dyslipidemia, elevated blood pressure and hyperglycemia, that markedly increases CVD risk (9). Such individuals represent a population in which combining genetic information with lifestyle monitoring could be particularly impactful, as an unfavorable polygenic architecture may synergize with metabolic and behavioral risk factors, thereby amplifying the lifetime cardiovascular risk trajectory and potentially accelerating the onset of major adverse cardiovascular events. Similarly, this population can take advantage of digital health technologies, such as wearable health devices (WHDs) capable of monitoring physical activity, dietary, and sleep habits giving the patient a feedback that could nudge him further toward healthy habits (10). Nevertheless, in order to facilitate the implementation of new health technologies and interventions, in addition to their efficacy currently under investigation in an ongoing trial (11), it is necessary to demonstrate their feasibility, considering factors that can prevent their adoption. These factors encompass among others, acceptability, demand, implementation, practicality (12), and their assessment can be useful for both refining methods to evaluate their efficacy and developing implementation strategies (13). To date, no study has evaluated whether PRS, alone or combined with WHDs, can be feasibly adopted within primary prevention programs for CVDs. The present paper describes the rationale and design of the FitPreV trial, a feasibility pilot randomized controlled study assessing the integration of PRS and WHDs in primary prevention for individuals with metabolic syndrome within the Italian National Health Service (SSN).

### General objective

The FitPreV study aims to evaluate the feasibility of implementing PRS and WHDs for CVD primary prevention within the SSN. Aligning with the PNRR's focus on proximity healthcare (Mission 6, Component 1) (14) the trial will assess their integration in General Practitioners' (GPs) practices. The study will target individuals with metabolic syndrome, a population at substantially increased CVD risk and potentially receptive to lifestyle interventions, with the goal of assessing the acceptability, practicality, and barriers to adoption of these innovative tools in a real-world primary care setting.

### Primary objective

Evaluate the feasibility of implementing PRS and WHDs in personalized cardiovascular prevention prompted by GPs.

### Secondary objectives

The secondary objectives include a preliminary assessment of the clinical impact of PRS and WHDs on cardiovascular prevention (15).

## Methods

This protocol is written according to the SPIRIT statement (16, 17).

The feasibility study employs a randomized controlled design with four parallel arms in a cohort of patients residing in the metropolitan area of Rome. Participants will be randomly allocated to one of the following intervention groups:

° The first group will receive standard medical counseling (Control group).
° The second group will receive standard counseling along with a wearable device and corresponding app (Wearable device group).
° The third group will receive standard counseling plus genetic risk information (PRS) obtained from saliva samples (Genetic risk group).
° The fourth group will receive standard counseling, genetic risk information and a wearable device (Combined group).

A detailed explanation of each of the interventions can be found in the “Interventions” section.

### Trial population and setting

The population will be composed of 120 participants aged 40 to 69 years with a diagnosis of metabolic syndrome and a 10-year CVD risk between 2.5 and 10%, who are under the care of GPs in Rome metropolitan area 2025–2026 (Italy).

### Inclusion criteria

Participants must meet the following criteria:

- Aged 40–69 years.
- SCORE2 10-year cardiovascular risk of 2.5%−10%.
- Diagnosis of metabolic syndrome according to American Heart Association (AHA) criteria, chosen for feasibility in primary care as it uses routinely collected data^9^.Specifically AHA criteria require the presence of at least three of the following:
  ° Central obesity (waist circumference greater than 102 cm in men or greater than 89 cm in women).
  ° Elevated triglycerides (≥150 mg/dL) or treatment for hypertriglyceridemia.
  ° Low HDL cholesterol (less than 40 mg/dL in men or less than 50 mg/dL in women) or treatment for dyslipidemia.
  ° Elevated blood pressure (systolic ≥130 mmHg or diastolic ≥85 mmHg).
  ° Elevated fasting blood glucose (≥100 mg/dL) or treatment for hyperglycemia.

### Exclusion criteria

Individuals with any of the following conditions will be excluded from the study:

- Diabetes mellitus.
- Familial hypercholesterolemia.
- Previous cardiovascular events.

### Primary endpoints

The feasibility indicators as detailed in their section.

### Secondary endpoints

Lifestyle changes measured through the LF8 questionnaire (18), changes in cardiovascular risk profiles evaluated through SCORE2 (15), biochemical variations including glucose, total cholesterol, HDL cholesterol, LDL cholesterol, triglycerides and glycated hemoglobin (HbA1c).

### Study procedures

**Figure 1:**
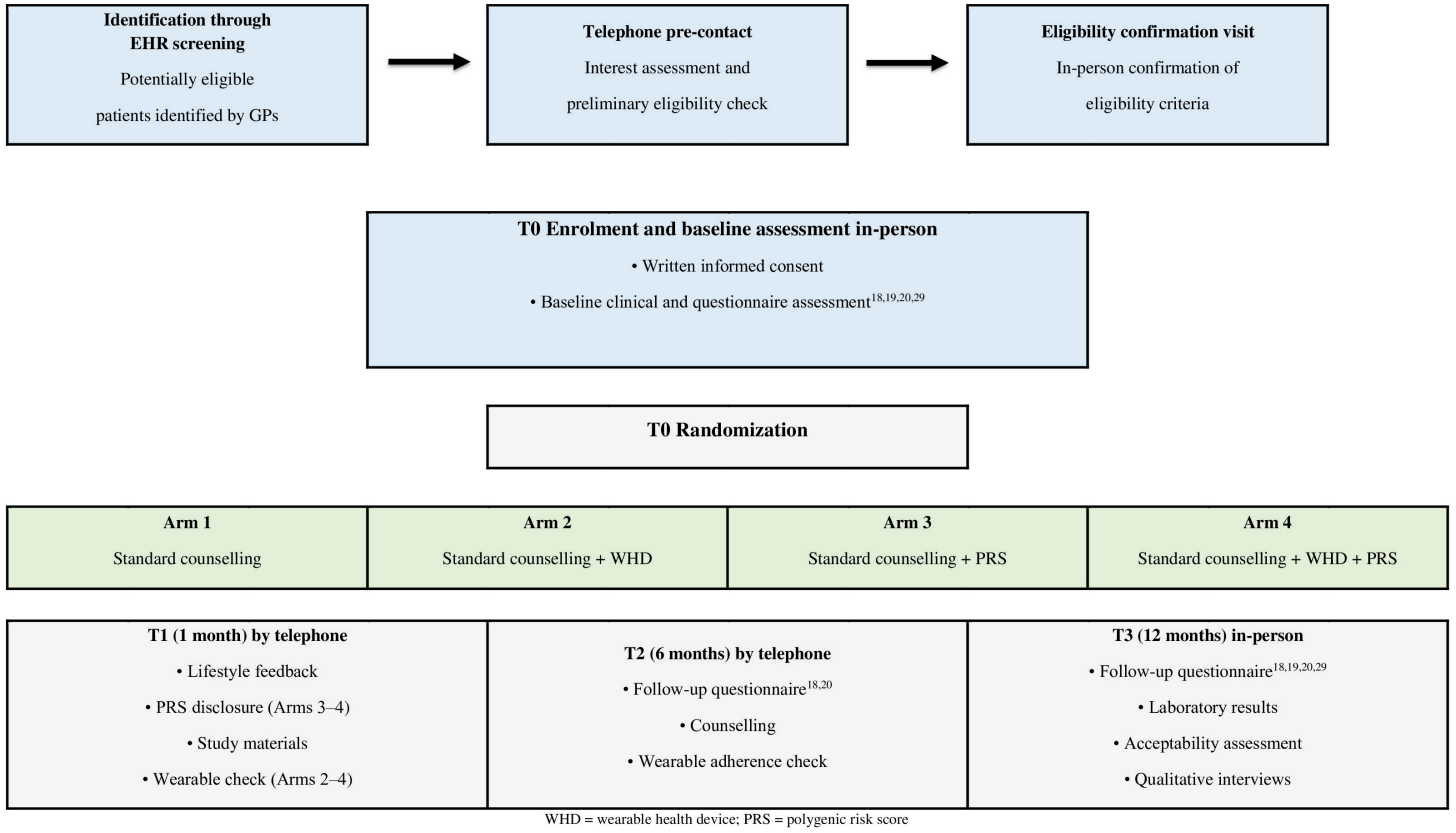
Study flow and timeline of the Fitprev pilot feasibility trail. WHD, wearable health device; PRS, polygenic risk score.

The study procedures will follow a structured timeline involving multiple assessments and interventions (see Figure 1).

#### Pre-screening

GPs will generate a preliminary list of eligible patients by querying their electronic health records (i.e., the practice's digital management systems for clinical data). Potentially eligible individuals will be contacted and invited to their GP's practice, where the study will be explained in detail. At the in-person visit, an additional verification of the inclusion and exclusion criteria will be performed.

#### Enrolment (T0)

GPs will provide verbal and written informed consent to eligible individuals, including specific modules for genetic analysis. Then, participants will be formally enrolled and randomly assigned to one of the four study arms using a computer-generated randomization procedure implemented through the REDCap (Research Electronic Data Capture) system, which will also record all participants information during the study. The assignment to the intervention will not be blinded. Participants, research staff, and the medical team will become aware of study group allocation only after completion of the randomization procedures. Given the nature of the intervention, blinding of participants and healthcare professionals is not feasible, as participants will know whether they receive PRS information and/or access to the app and wearable device. Outcome assessment cannot be fully blinded, particularly for self-reported behavioral outcomes, because these outcomes are directly provided by participants who are aware of their allocation. Standardized study materials and instructions will be provided to GPs to support consistency in intervention delivery across study arms.

All participants will undergo a comprehensive assessment and cardiovascular evaluation. This will include the completion of questionnaires covering socioeconomic status, area of residence and key lifestyle factors such as smoking, alcohol consumption, dietary patterns, sleep and physical activity. Cardiovascular health will be assessed using the Life's Essential 8 (LE8) framework (18) developed by the American Heart Association (AHA), which encompasses eight domains: physical activity, nicotine exposure, sleep health, body mass index, blood lipids, blood glucose, blood pressure and diet (Annex 1, section 1, section 2, section 3). Each component is scored on a continuous scale from 0 to 100 according to AHA criteria. The overall LE8 score is calculated as the unweighted mean of the eight components, resulting in a composite score ranging from 0 to 100, with higher values indicating more favorable cardiovascular health. In line with AHA classification, scores will be categorized as poor (0–49), intermediate (50–79), or ideal (80–100). Dietary quality will be assessed using the modified Mediterranean Eating Pattern for Americans (MEPA) (19). Based on the LE8 assessment, participants will be categorized into favorable, intermediate, or unfavorable lifestyle profiles.

Additionally, participants will be asked questions to assess their perceptions of cardiovascular risk and the perceived benefits of preventive interventions (20). All questionnaires are provided in Appendix 1.

For participants assigned to groups 3 and 4, saliva samples will be also collected to conduct PRS analysis to evaluate their genetic risk profile for CVD.

For participants in groups 2 and 4, wearable devices will be provided along with instructions on how to set up and use the accompanying mobile application.

#### T1 (1 month post-enrolment)

All participants will be informed about their lifestyle profile through direct communication, including telephone contact, and will receive written materials via email outlining standard behavioral interventions to reduce cardiovascular risk, regardless of group assignment. Participants in groups 3 and 4 will receive detailed explanations of their genetic risk results derived from the PRS analysis. This will include information about their categorized risk level and the implications for their cardiovascular health, helping them understand how their genetic predisposition may affect their overall risk.

#### T2 (6 months post-enrolment)

All participants will be contacted by GPs by telephone for a follow-up assessment where they will complete the questionnaire to monitor changes in their health behaviors. Participants will be extemporarily informed of their progress and any updated lifestyle classification. Moreover, extra counseling and support will be provided to reinforce positive lifestyle changes.

#### T3 (12 months post-enrolment)

Participants will return to their GPs for a comprehensive assessment, completing a final lifestyle questionnaire. Participants will undergo blood tests to re-evaluate total cholesterol, HDL, LDL, triglycerides, glucose, and hemoglobin A1c levels. Moreover, participants will complete a questionnaire designed to evaluate the feasibility and acceptability of the interventions, including their experiences with the innovative interventions and their perceived utility. At T3, the GPs will also complete a questionnaire to assess the feasibility of the interventions from their perspective (Annex1, Section 7). Additionally, a sample of both GPs and patients will participate in qualitative interviews to provide insights on their experiences and observations throughout the study. A convenience sampling will be adopted to identify GPs and patients for the interview. The sampling will be done separately for the *four* trial arms trying to have a balance with respect to sex. A thematic framework will be used to develop the guide for the interviews, and the latter will be conducted by a group of trained professionals and recorded.

## Interventions

### Genetic testing

Genotyping is planned for participants in groups 3 and 4 to support the calculation of of a commercially available genome-wide PRS, provided by an external provider. Genotyping will be performed using Illumina microarray technology (21), a cost-effective platform for large-scale SNP profiling in disease and population genetics studies. This approach generates data on millions of genetic variants per individual. Missing genotypes will be imputed using a modified version of the GLIMPSE algorithm (22), leveraging reference panels from the 1,000 Genomes Project (23) to ensure high-quality genome-wide coverage.

The PRS was developed and validated in large, ancestrally diverse cohorts (totaling over 29,000 individuals across multiple genetic ancestry groups, including European, African, South Asian, and others), demonstrating robust predictive performance (odds ratio per standard deviation of approximately 1.57) (24).

The resulting CVD genetic risk profile will be communicated to clinicians (and subsequently to participants) using a three-tier classification based on the participant's PRS percentile rank within the population distribution:

- **Normal risk:** PRS below the 75th percentile.
- **Moderate risk:** PRS between the 75th and 97th percentiles (approximately 2-fold increased risk compared to the population average).
- **High risk:** PRS at or above the 97th percentile (top 3% of the population; approximately 3-fold increased risk compared to the population average).

### Wearable devices

Participants assigned to groups 2 and 4 will receive a short written user guide and will be instructed to use a wearable device in combination with a paired smartphoneapplication (25). The app is available for both Android and iOS devices and requires installation on a compatible smartphone, creation of a personal account and Bluetooth pairing with the wearable. The app provides consumer-facing health and activity indicators, including step count, sleep, heart rate, blood oxygen saturation, calories burned, and stress level, and also supports exercise session tracking. Additional technical characteristics relevant to study implementation are reported in Annex 2, Supplementary Table 1.

Use of the wearable device will not be actively monitored by the study team, and no data will be collected to assess adherence to device use.

### Data collection and management

Data will be captured using REDCap (Research Electronic Data Capture), a secure, web-based application designed for research data collection, ensuring data integrity, consistency, and confidentiality. Access to the REDCap platform and to data entry and management functions will be restricted to authorized study investigators and data managers. Each GP will enter data into electronic case report forms (eCRFs) in pseudonymized form.

All data collected within the FitPreV study will be managed in compliance with the General Data Protection Regulation (EU 2016/679). No data from wearable devices will be directly collected; their use will be assessed *post hoc* through self-report questionnaires. Prior to enrolment, all participants will provide written informed consent, including a specific section addressing the use of genetic data. Outcome data will be collected using standardized case report forms and questionnaires across all study arms.

Particular attention will be given to the governance of PRS-related information, including the secure handling of genetic reports, controlled communication of risk information, and restricted access to identifiable data.

### Statistical analysis

Randomization will be performed using a computer-generated sequence with randomly permuted blocks with random block sizes and block order. Allocation sequences will be securely stored, and participants will be assigned to their study group after a 1:1:1:1 randomization. The arm assignment will be centrally provided through REDCap platform.

As this is a feasibility study and not designed to assess clinical efficacy, no formal power calculation has been performed, in line with the CONSORT extension for randomized pilot and feasibility trials (26). The sample size has instead been determined based on practical considerations and the need to assess key feasibility outcomes, including recruitment, retention, adherence, and data completeness. While no formal minimum sample size is established for feasibility studies, small sample sizes have been suggested in the literature (27). A total of 120 participants is considered sufficient to provide informative estimates of these parameters and to support the design of a future definitive trial (28).

Feasibility indicators will be summarized using descriptive statistics: absolute and relative frequencies for categorical variables and means with standard deviations or medians with interquartile ranges for continuous variables. Where appropriate, 95% confidence intervals will be reported. Missing data patterns will be described without imputation, as recommended for feasibility designs. Feasibility outcomes will also be summarized at the GP level to explore variability in recruitment, data completeness, and acceptability across practices.

Exploratory analysis will be conducted to provide preliminary insights into potential effects of the interventions on lifestyle behaviors, lipid profiles, and cardiovascular risk. For this purpose, the three intervention groups will be compared with the control group to estimate the marginal effect of each intervention and their combination. Mixed-effects models for repeated measures will be employed to assess within- and between-group differences over time for continuous outcomes, such as lifestyle scores, lipid levels, and SCORE2 cardiovascular risk estimates. These models will include fixed effects for group, time, and their interaction, and random effects for participants to account for intra-individual correlation. Given that participants are nested within GPs, potential GP-level clustering will be explored by including GP as a random effect, where appropriate. Models will adjust for baseline values and stratification factors. Effect estimates will be presented as mean differences with 95% confidence intervals, recognizing that the study is not powered for hypothesis testing. Effect estimates will be presented as mean differences with corresponding 95% confidence intervals. Given the exploratory nature of the analyses, emphasis will be placed on the magnitude and precision of the estimates rather than on formal hypothesis testing. All analyses will be performed using Stata (StataCorp. 2025. Stata Statistical Software: Release 19. College Station, TX: StataCorp LLC) and R (https://www.r-project.org/). In respect to the qualitative interviews, recordings will be analyzed using thematic analysis through a double-coding approach.

### Feasibility indicators

Feasibility indicators are selected in line with commonly described domains in implementation research, including aspects of perceived usefulness, burden, and overall participant experience. In detail, to quantify the feasibility of the intervention, several indicators will be assessed:

1. Structural readiness
  1.1 Experience of GPs in Clinical Studies: Prior experience of participating GPs in clinical research (Annex 1, section 7).
  1.2Availability of Necessary Tools and Facilities: This indicator assesses whether the general practices have the required resources to implement the interventions (Annex 1, section 7).
2. Process feasibility
  2.1 Recruitment rates: Proportion of participants who are successfully recruited for the study relative to the total number of eligible patients.
  2.2 Retention rates: Proportion of enrolled participants who remain in the study throughout its duration.
  2.3 Attendance rates: Proportion of scheduled sessions that participants attend compared to the total number of sessions they were expected to attend.
3. Acceptability
  3.1 Subjective Perceptions: Participants and physicians' perceptions of the intervention's feasibility and acceptability (20) (Annex 1, section 6).
  Validated questionnaires will assess participants' perceptions regarding:
  - Their understanding of cardiovascular risk.
  - Perceived severity of CVD.
  - Perceived benefits of the interventions.
  - Perceived barriers to participation.
  - Perceived benefits and barriers regarding the Polygenic Risk Score.
  Technology Acceptance Model (TAM) (29, 30): To evaluate the acceptability of wearable devices, participants will respond to questions based on the TAM (Annex 1, section 5) such as
  - Ease of use of the devices.
  - Usefulness of the devices in managing their health.
  - Usefulness of the information regarding the Polygenic Risk Score.
  - Intention to continue using the devices beyond the study period.
4. Burden
  Burden: The perceived burden of participation among participants and physicians. Both will be asked to evaluate whether the intervention's objectives are clear and if the time and effort needed for implementation are manageable within their daily routine (Annex 1, section 6 and 7).
5. Participant capability
  Self-Efficacy: Participant's beliefs in their ability to engage with the interventions effectively. Participants will respond to questions about their confidence in making lifestyle changes and managing their cardiovascular risk based on the information and tools provided during the study (Annex 1, section 6).

### Criteria for progression toward a full trial

In line with methodological recommendations for feasibility studies, which emphasize the importance of prospectively defined progression criteria, this study includes predefined thresholds to guide decisions regarding progression to a future definitive trial (26, 31).

The following *a priori* progression criteria have been defined:

(a) **Recruitment:** At least 30% of eligible participants agree to participate and are randomized.

(b) **Completeness of outcome data:** Outcome data are available for at least 90% of participants, as recorded in the eCRFs.

(c) **GPs' willingness to participate:** At least 70% of participating GPs report that they would be willing to implement the study in routine practice.

## Discussion

This protocol outlines one of the first initiatives to assess the feasibility of integrating PRS and wearable devices into primary cardiovascular prevention within the context of a public healthcare system. The study is designed to contribute to the growing body of evidence supporting the potential role of personalized prevention strategies in mitigating CVD risk. Ongoing trials, such as INNOPREV (11), are primarily focused on evaluating the clinical efficacy of PRS-based interventions, while evidences on their feasibility, acceptability, and implementation in real-world settings remains scarce. FitPreV aims to address this gap by generating critical evidence on operational workflows, organizational readiness, and user acceptability that will inform the scalable integration of PRS and wearable technologies into routine primary care.

Previous studies have shown that both patients and healthcare professionals generally perceive PRS-based tools as acceptable and potentially useful for clinical decision-making. For instance, the CVD-IRT pilot in the UK showed that integrating PRS with the QRISK2 algorithm during routine health checks was feasible and well accepted: over 90% of general practitioners considered the integration straightforward, and 86.9% of patients expressed willingness to recommend it (32). Similarly, Viigimaa et al. (33) reported that disclosing high polygenic risk for coronary artery disease influenced physician prescribing behavior and increased statin initiation in primary care. These studies underline that PRS information can meaningfully affect risk perception and management strategies.

Our study differs from these experiences in two main aspects. First, it combines genetic risk stratification with continuous lifestyle monitoring through wearable technology, reflecting a holistic approach to personalized prevention. Second, it evaluates these strategies within the Italian NHS, a universal, GP-centered system that differs substantially from the more fragmented healthcare structures studied so far. This design allows exploration of organizational and acceptability-related challenges that are critical to scaling up personalized prevention in a public health setting. Moreover, a key strength of FitPreV is its focus on a high-risk population: individuals with metabolic syndrome. Combining PRS and wearable-based lifestyle monitoring in this group is particularly relevant, as genetic susceptibility may synergize with metabolic and behavioral risk factors, accelerating the trajectory of cardiovascular risk. Evaluating feasibility in this population ensures that findings are highly informative for real-world implementation in those who stand to benefit most.

Anticipated challenges, in line with prior literature (34, 35), include the lack of standardized clinical guidelines for PRS interpretation, the need for GP training, and the complexity of communicating polygenic risk in a way that is both comprehensible and actionable. Stakeholders also report variability in familiarity, confidence, and willingness to use PRS in clinical practice, emphasizing the need for clear frameworks and decision-support tools to facilitate implementation.

Similarly, for wearable health devices, several user- and system-level barriers have been documented. Common reasons for device discontinuation include difficulties with daily use, non-intuitive interfaces, limited battery life, as well as a perceived lack of tangible or immediate benefit (36, 37). Importantly, acceptability of WHDs is not uniform across patients: recruited individuals with chronic conditions may be more inclined to use such technologies, but they may also express heightened concerns about potential negative consequences (38).

From a policy standpoint, these innovations align with current European health priorities, including the Italian PNRR (39), which emphasizes proximity care and the integration of digital health tools. However, several structural and methodological challenges could affect the broader implementation of PRS-based prevention. First, the clinical utility of PRS remains to be fully shown, as prospectively collected outcome data are still limited despite strong evidence of genetic associations with common diseases. Although FitPreV is not powered to assess clinical efficacy, it will provide exploratory data on intermediate outcomes, such as changes in cardiovascular risk profiles and lipid levels, that can inform trials designed to establish clinical benefit. Second, primary care, where most preventive interventions are delivered, is typically time- and resource-constrained, making the integration of PRS and digital tools challenging without streamlined workflows and robust decision-support systems. Addressing this operational issue is one of the key objectives of FitPreV. Finally, although methodological advances and increasingly diverse cohorts have improved PRS prediction, concerns remain regarding their limited validity in underrepresented populations and the risk of exacerbating health disparities if these tools are adopted without adequate safeguards.

Despite these strengths, several limitations should be considered when interpreting the findings. First, the lack of blinding is inherent to the nature of the intervention. Participants, healthcare professionals, and research staff cannot be blinded to group allocation, and outcome assessment cannot be fully blinded, particularly for self-reported behavioral outcomes. Consequently, lifestyle-related outcomes such as diet, physical activity, smoking, alcohol consumption, and sleep habits may be affected by reporting, social desirability, or performance bias. To mitigate these risks, standardized questionnaires and data collection procedures will be used across all study arms, and objective clinical, anthropometric, and laboratory measures will be collected where applicable to complement self-reported outcomes. Blinding during data analysis will also not be feasible due to the collection of arm-specific variables; therefore, analyses will be conducted using coded participant identifiers and predefined statistical analysis procedures to reduce the risk of analytical bias. Wearable-generated data and app-level usage logs will not be directly collected for research purposes, and adherence will therefore be assessed through participant self-report, which may be subject to recall bias and inaccurate reporting. However, the wearable is intended primarily as a supportive tool to promote behavioral change rather than as a primary outcome measure. In addition, participants are nested within general practitioners, and although standardized materials are used to support consistent intervention delivery, some degree of clustering and potential contamination across study arms cannot be excluded. The study does not include a prospective economic evaluation or systematic assessment of implementation costs, limiting conclusions regarding cost-effectiveness and scalability. Finally, the qualitative component is based on voluntary participation, which may introduce self-selection bias, with more motivated individuals potentially over-represented.

Ultimately, by systematically addressing feasibility before moving to large-scale trials, FitPreV aims to bridge the gap between innovation and implementation, paving the way for the integration of personalized prevention strategies, such as PRS and wearable technologies, into routine cardiovascular care within public health systems. FitPreV will provide critical insights into the challenges and facilitators of adopting PRS and wearable technologies in primary prevention. This pragmatic approach reduces the risk of failure in subsequent effectiveness trials and ensures that future research is grounded in real-world applicability. These results will inform the design of a definitive multicenter trial evaluating clinical outcomes in similar on-field conditions.
