# Supplementary material for: Feasibility of integrating genetic risk and digital health tools for cardiovascular prevention: the FitPreV protocol
Source: Front Public Health. 2026 May 29;14:1800961. doi: 10.3389/fpubh.2026.1800961 (PMC13260590; doi:10.3389/fpubh.2026.1800961)
Supplement: Supplementary file 1 [file Table_1.docx]

| **WHD characteristics** | **Description** |
| --- | --- |
| Smartphone compatibility | Android and iOS |
| Initial setup | The device is activated by charging and configured through language selection, followed by QR-code-based download of the companion app and Bluetooth pairing with the smartphone |
| User account requirements | Users are required to install the app, accept terms and conditions, select the region and create a personal account |
| Connectivity | Bluetooth connection between the wearable device and smartphone |
| User-enabled monitoring functions | App notifications, heart rate monitoring, sleep monitoring and blood oxygen monitoring |
| User-facing health/activity metrics | Steps, sleep, heart rate, blood oxygen saturation, calories burned and stress level |
| Exercise-related functions | Outdoor running, walking, outdoor cycling and treadmill workout sessions |
| Wearing instructions | The device is intended to be worn on the wrist, with specific instructions provided for routine use and physical activity to ensure adequate skin contact for optical heart rate sensing |
| Charging requirements | Magnetic charging base connected to a phone charger or PC USB port |
| Data visualization | Data are displayed in the “Health” section of the companion app |
| Validation status | No study-specific validation of step count, heart rate, sleep or blood oxygen saturation measures was performed |
| Sampling frequency | Sampling frequency was not available from the user-facing setup guide and was not directly accessible within the study procedures |
| Technical requirements | Smartphone, app installation, personal account creation, Bluetooth activation and internet access for app download and account registration |
| Data ownership/access in the study | Device- and app-generated data were not directly extracted by the research team; study assessments were based on participant self-report |
| Integration with study database | No automatic integration with REDCap or other study databases was implemented |

Table 1. Wearable device and companion app characteristics
