# Supplementary material for: Feasibility of integrating genetic risk and digital health tools for cardiovascular prevention: the FitPreV protocol
Source: Front Public Health. 2026 May 29;14:1800961. doi: 10.3389/fpubh.2026.1800961 (PMC13260590; doi:10.3389/fpubh.2026.1800961)
Supplement: Supplementary file 2 [file Table_2.docx]

**FitPreV Questionnaire**

**SECTION 1. DEMOGRAPHIC INFORMATION, RESIDENTIAL AREA, AND FAMILY HISTORY OF CARDIOVASCULAR RISK**

*Lloyd-Jones, D. M. et al. Life’s Essential 8: Updating and Enhancing the American Heart Association’s Construct of Cardiovascular Health: A Presidential Advisory from the American Heart Association. Circulation 146, E18–E43 (2022).*

**Demographic and Residential Information**

**1.a) Personal Data**

- Sex: M or F
- Age (in years)
- Citizenship: Italian or other (if other, specify)
- City:
- Enter your ZIP code:

**1.b) What is your highest level of education attained?**

- No formal education
- Primary school diploma
- Lower secondary school diploma
- Upper secondary school diploma
- Bachelor’s degree
- Master’s degree
- PhD
- Prefer not to answer

**1.c) What is your marital status?**

- Single
- Engaged
- Cohabiting
- Married
- Separated/divorced
- Widowed
- Prefer not to answer

**1.d) Where do you live?**

- In a large city (more than 200,000 inhabitants)
- In a city (fewer than 200,000 inhabitants)
- In a rural area (e.g., countryside, mountains)
- Prefer not to answer

**1.e) What is your employment status?**

- Full-time employment
- Working fewer than five days per week
- Part-time employment
- Disability pensioner
- Retirement pensioner
- Partially retired
- Unemployed
- Homemaker
- Other (specify)
- Prefer not to answer

**Family History**

**[Has any of your family members (parents, siblings, children) experienced one or more of the following conditions?]**

**1.f) Premature cardiovascular diseases (heart attack, angina, cardiac ischemia, sudden cardiac death, angioplasty (balloon/stent), or coronary bypass), respond:**

- No
- Yes
- Don’t know

**1.g) Premature cerebrovascular diseases (stroke, hemorrhagic stroke, cerebral ischemia, transient ischemic attack (TIA)), respond:**

- No
- Yes
- Don’t know

**[Among your family members, has anyone experienced:]**

**1.h) High cholesterol (in parents, siblings, children)**

- No
- Yes
- Don’t know

**1.i) Diabetes (in at least one biological parent)**

- No
- Yes
- Don’t know

**1.j) Diabetes (in siblings, children)**

- No
- Yes
- Don’t know

**1.k) Diabetes (in grandparents, uncles/aunts, cousins)**

- No
- Yes
- Don’t know

**1.l) Hypertension (father)**

- No
- Yes
- Don’t know

**1.m) Hypertension (mother)**

- No
- Yes
- Don’t know

**SECTION 2. TYPES OF FOODS INCLUDED IN YOUR DIET (LF8)**

Any number greater than or equal to 0 is allowed.

**2.a) How many tablespoons of olive oil do you consume per day (including oil used for seasoning and cooking)?**

**2.b) How many servings (approximately 200 g per serving) of vegetables do you consume per day?**

**2.c) How many servings of fruit (approximately 150 g per serving, e.g., one apple) do you consume per day?**

**2.d) How many servings of bread, pasta, rice, or cereals do you consume per day? (One serving of bread = 50 g; pasta or cereals = 80 g)**

**2.e) How many times per day do you consume sweets (e.g., cookies, pastries, cake slices, croissants, brioches)?**

**2.f) How many servings (approximately 80 g per serving) of leafy greens (e.g., spinach) do you consume per week ?**

**2.g) How many servings (approximately 80 g per serving) of berries do you consume per week ?**

**2.h) How many servings (approximately 100 g per serving) of red meat do you consume per week ?**

**2.i) How many servings (approximately 150 g per serving) of fish (not fried) do you consume per week ?**

**2.j) How many servings (approximately 100 g per serving) of chicken (not fried) do you consume per week ?**

**2.k) How many servings of cheese (at least 50 g per serving) do you consume per week ?**

**2.l) How many servings (approximately 10 g per serving) of butter do you consume per week ?**

**2.m) How many servings of fresh or canned legumes (approximately 150 g) or dried legumes (approximately 50 g) do you consume per week ?**

**2.n) How many servings (approximately 30 g per serving) of nuts (e.g., almonds, hazelnuts, walnuts) do you consume per week ?**

**2.o) How many meals do you consume at fast-food restaurants (e.g., McDonald’s or Burger King) per week ?**

**SECTION 3. LIFESTYLE (PHYSICAL ACTIVITY, SMOKING, ALCOHOL CONSUMPTION, SLEEP DURATION) AND LIPID-LOWERING THERAPY**

**Physical Activity**

**3.a) How many minutes of vigorous-intensity physical activity (e.g., weightlifting, heavy gardening, aerobic activities like running or cycling at a fast pace) do you perform per week ?**
Any number greater than or equal to 0 is allowed(in minutes).

**3.b) How many minutes of moderate-intensity physical activity (e.g., carrying light loads, cycling at a regular pace, gym workouts, gardening, prolonged housework, brisk walking) do you perform per week ?**
Any number greater than or equal to 0 is allowed (in minutes).

**NB: In the final score, these two questions will be combined.**

**Smoking (Nicotine Exposure)**

**3.c) Are you a smoker?**

- **Never been a smoker**
- **Currently a smoker:** (if yes, answer the following)
  - **How many cigarettes per day?** (a number greater than 0)
  - **For how many years?** (a number greater than 0)
    - **Type:**
      - Heated tobacco cigarettes (e.g., Iqos, Glo)
      - E-cigarettes
      - Other (specify)
- **Former smoker:** (if yes, open the following fields)
  - **Less than one year ago**
  - **1–<5 years ago**
  - **More than five years ago**
    - **Type:**
      - Heated tobacco cigarettes (e.g., Iqos, Glo)
      - E-cigarettes
      - Other (specify)

**Alcohol Consumption**

**3.d) How many alcohol units do you consume per day ?** One alcohol unit corresponds to 12 grams of pure alcohol and is equivalent to:

- One glass of wine (125 ml at 12%)
- One can of beer (330 ml at 4.5%)
- One aperitif (80 ml at 38%)
- One shot of spirits (40 ml at 40%)

Any number greater than or equal to 0 is allowed.

**Sleep Duration**

**3.e) On average, how much do you sleep in a [24-hour] period?**

Any number greater than or equal to 0 is allowed.

**Lipid-Lowering Therapy**

**3.f) Are you taking medications for your lipid profile (e.g., statins, fibrates, red yeast rice supplements)?**

- No
- Yes
- If yes, specify which and the daily dosage.

**SECTION 4. LABORATORY AND CLINICAL PARAMETERS OF THE PATIENT**

**Blood Pressure (in mmHg)**

**4.a) Systolic blood pressure:**

**4.b) Diastolic blood pressure:**

**4.c) Are you taking medications to lower blood pressure?**

- No
- Yes (if yes, specify which, open-ended response)

**Cholesterol (Total, HDL, Non-HDL) and Triglycerides (in mg/dL)**

**4.d) Date of last test** (dd/mm/yy)

**4.e) Total cholesterol**

**4.f) HDL cholesterol**

**4.g) Non-HDL cholesterol** (non-high-density lipoprotein cholesterol, calculated as total cholesterol minus HDL cholesterol)

**4.h) Triglycerides**

**4.i) Are you taking medications to lower cholesterol?**

- No
- Yes (if yes, specify which, open-ended response)

**Blood Glucose (mg/dL) or HbA1c (%)**

**4.j) Date of last test** (dd/mm/yy)

**4.k) Do you have diabetes?**

- No
- Yes

**4.l) Fasting blood glucose (mg/dL):**

**4.m) Glycated hemoglobin HbA1c (%):**

**Body Mass Index**

**4.n) Height (in cm):**

**4.o) Weight (in kg):**

**Based on these values, the BMI will be automatically calculated.**

**4.q) Waist circumference (in cm):**

**SECTION 5. QUESTIONNAIRE ON PERCEPTIONS, KNOWLEDGE, ATTITUDES, AND BEHAVIORS IN CARDIOVASCULAR HEALTH AND STUDY TECHNOLOGIES.**

*Stubbs, D., Hooker, G. W., Li, Y., Richter, L. & Bick, A. Development and validation of the Vanderbilt PRS-KS, an instrument to quantify polygenic risk score knowledge. Genetics in Medicine Open 1, 100822 (2023).*

*Haydon, H. M. et al. Development and validation of the Digital Health Acceptability Questionnaire. J. Telemed. Telecare 29, 8S-15S (2023).*

**Reaction to Genetic Testing**

**If you received a genetic test result indicating a higher risk of cardiovascular diseases...**

**5.a) I would feel there is nothing I can do to prevent cardiovascular diseases:**

- Not at all
- A little
- Moderately
- Very
- Extremely

**5.b) I would be worried about having a cardiovascular disease:**

- Not at all
- A little
- Moderately
- Very
- Extremely

**5.c) I would regret taking the test:**

- Not at all
- A little
- Moderately
- Very
- Extremely

**5.d) I would feel depressed about the result:**

- Not at all
- A little
- Moderately
- Very
- Extremely

**5.e) I would be happy to know the result:**

- Not at all
- A little
- Moderately
- Very
- Extremely

**5.f) Have you discussed your cardiovascular disease risk with your family or friends?**

- No, I haven’t talked to anyone.
- Yes, I’ve had conversations with them but haven’t encouraged them to consult a doctor.
- Yes, I’ve had conversations with them and encouraged them to consult a doctor.
- Yes, after talking with me, someone I know has seen a doctor.

**Technologies**

**5.g) How would you rate your computer skills?**

- Poor
- Fair
- Adequate
- Good
- Excellent

**5.h) Do you use the following devices, and if so, primarily for what purpose?**

- Computer:
  - Yes/No
  - If yes:
    - Work
    - Leisure
- Tablet:
  - Yes/No
  - If yes:
    - Work
    - Leisure
- Smartphone:
  - Yes/No
  - If yes:
    - Work
    - Leisure

**5.i) Have you ever used digital health applications (apps)?**

- Yes
- No

**5.j) HBCVD**

*Tovar EG, Rayens MK, Clark M, Nguyen H. Development and psychometric testing of the Health Beliefs Related to Cardiovascular Disease Scale: preliminary findings. J Adv Nurs. 2010 Dec;66(12):2772-84. doi: 10.1111/j.1365-2648.2010.05443.x. Epub 2010 Sep 10. PMID: 20831570.*

**All items require a single response on a Likert scale.**

**[1 = strongly disagree; 2 = disagree; 3 = agree; 4 = strongly agree]**

**Category: Susceptibility**

1. It is likely that I will have a heart attack or stroke in the future.
2. My chance of having a heart attack or stroke in the next few years is high.
3. I believe I will have a heart attack or stroke at some point in my life.
4. It is currently possible that I could have a heart attack or stroke.
5. I am worried about having a heart attack or stroke in the near future.

**Category: Severity**

1. Having a heart attack or stroke always leads to death.
2. Having a heart attack or stroke would harm my relationships with loved ones.
3. My whole life would change if I had a heart attack or stroke.
4. Having a heart attack or stroke would have a very negative effect on my sex life.
5. If I had a heart attack or stroke, I would die within the next ten years.

**Category: Benefits**

1. Increasing physical activity reduces my chances of having a heart attack or stroke.
2. Following a healthy diet reduces my chances of having a heart attack or stroke.
3. Following a healthy diet and exercising for 30 minutes most days of the week is one of the best ways to prevent a heart attack or stroke.
4. If I exercise, I am doing something good for myself.
5. If I eat healthily, I am doing something good for myself.
6. Following a healthy diet reduces my chances of dying from cardiovascular diseases.

**Category: Barriers**

1. I don’t know the type of exercises most suitable for reducing my risk of cardiovascular diseases.
2. It is tiring for me to walk for more than five minutes.
3. I have access to facilities and/or equipment for physical activity.
4. I have someone willing to exercise with me.
5. I don’t have time to exercise for 30 minutes a day most days of the week.
6. I don’t know what constitutes a healthy diet to protect against cardiovascular diseases.
7. I don’t have time to cook.
8. I can’t afford to buy healthy foods.
9. I have more important problems than worrying about diet and exercise.

**5.k) The Vanderbilt PRS-KS**

*Stubbs D, Hooker G, Li Y, Richter L, Bick A, Development and Validation of the Vanderbilt PRS-KS, an Instrument to Quantify Polygenic Risk Score Knowledge, Genetics in Medicine Open (2023), doi:*[*https://doi.org/10.1016/j.gimo.2023.100822*](https://doi.org/10.1016/j.gimo.2023.100822)*.*

**[All items require a single TRUE/FALSE response.]**

1. The polygenic risk score is based on genetic changes present in multiple genes.
2. All individuals with a high polygenic risk score for a given disease will develop it.
3. The polygenic risk score can consider other health determinants besides genetics (e.g., ethnicity).
4. The polygenic risk score has the same accuracy regardless of the disease for which it is calculated.
5. The polygenic risk score has the same accuracy for all people, regardless of their racial or ethnic origin.
6. If a person’s polygenic risk score is high, their children’s will also be high.
7. The polygenic risk score could reveal a reduced risk of disease.

**SECTION 6. FEASIBILITY OF STUDY PROCEDURES FOR PATIENTS**

**Final Feasibility Questionnaire for the Patient**

Dear Patient,

Thank you for accompanying us on this journey. The following questionnaire should be completed at the end of the study.

Indicate how intensely the following operations influenced your emotions on a scale from 1 (Not at all) to 5 (Extremely):

| **Operation** | **1 (Not at all)** | **2 (A little)** | **3 (Moderately)** | **4 (Very)** | **5 (Extremely)** |
| --- | --- | --- | --- | --- | --- |
| Learning the results of the Polygenic Risk Score made me more anxious about my health. |  |  |  |  |  |
| Learning the results of my Polygenic Risk Score made me more proactive in my efforts to preserve my health. |  |  |  |  |  |
| Using the electronic bracelet made me more anxious about my health. |  |  |  |  |  |
| Using the electronic bracelet made me more proactive in my efforts to preserve my health. |  |  |  |  |  |

If you were asked to wear the electronic bracelet, for how long do you estimate you wore it?

- Less than 1/3 of the time
- Between 1/3 and 2/3 of the time
- More than 2/3 of the time

If during the study you were asked to wear the electronic bracelet, please indicate your level of agreement with the following statements:

| **Statement** | **1 (Strongly agree)** | **2** | **3** | **4** | **5** | **6** | **7 (Strongly disagree)** |
| --- | --- | --- | --- | --- | --- | --- | --- |
| I find the wearable device uncomfortable to use. |  |  |  |  |  |  |  |
| Learning to use the wearable device was easy. |  |  |  |  |  |  |  |
| Using the wearable device was often frustrating. |  |  |  |  |  |  |  |
| Using the wearable device improves my quality of life. |  |  |  |  |  |  |  |
| The wearable device gives me greater control over my lifestyle. |  |  |  |  |  |  |  |
| I intend to continue using the wearable device in the future. |  |  |  |  |  |  |  |
| I would recommend others to use the wearable device. |  |  |  |  |  |  |  |

How much do you agree with the following statement?

| **Statement** | **1 (Not at all)** | **2 (A little)** | **3 (Neutral)** | **4 (Quite a bit)** | **5 (Completely)** |
| --- | --- | --- | --- | --- | --- |
| I believe that by participating in the study, my lifestyle has become healthier. |  |  |  |  |  |

**SECTION 7. FEASIBILITY OF STUDY PROCEDURES FOR GENERAL PRACTITIONERS (GPs)**

**Final Feasibility Questionnaire for General Practitioners**

Dear Colleague,

Thank you for accompanying us throughout the duration of the study. The following questionnaire should be completed at the end of the study, i.e., when the follow-up of all your patients has concluded.

For each of the following statements, please indicate with a cross how much you agree from 1 (Not at all) to 5 (Completely).

| **Statement** | **1 (Not at all)** | **2 (A little)** | **3 (Neutral)** | **4 (Quite a bit)** | **5 (Completely)** |
| --- | --- | --- | --- | --- | --- |
| The tools (tubes for saliva collection, electronic bracelets) necessary for the study were provided on time and in adequate quantities. |  |  |  |  |  |
| It was clearly explained to me how to administer the patient questionnaire. |  |  |  |  |  |
| It was clearly explained to me how to complete the electronic medical record on REDCap. |  |  |  |  |  |
| It was easy for me to complete the electronic medical record on REDCap. |  |  |  |  |  |
| The new procedures introduced by the study (Polygenic Risk Score) could become part of my daily clinical practice. |  |  |  |  |  |
| The objectives, rationale, and activities of the study were clear and understandable to me. |  |  |  |  |  |
| The activities required by the study posed a problem in the normal routine of my work. |  |  |  |  |  |
| The timeframes and commitment required by the study activities were acceptable. |  |  |  |  |  |

Have you previously participated in research projects or scientific studies?

- Yes
- No
- Don’t know/Uncertain

In one month, how many patients meeting the study inclusion criteria (*intermediate cardiovascular risk according to SCORE2, age 40–69, metabolic syndrome, no diabetes, no familial hypercholesterolemia*) visited your practice?
……………………………………………………………………………………………………………………

Indicate how difficult you found each of the following study operations, from 1 (Not at all) to 5 (Extremely):

| **Operation** | **1 (Not at all)** | **2 (A little)** | **3 (Moderately)** | **4 (Very)** | **5 (Extremely)** |
| --- | --- | --- | --- | --- | --- |
| Finding patients who meet the inclusion criteria. |  |  |  |  |  |
| Explaining and convincing the patient to participate in the study. |  |  |  |  |  |
| Ensuring the questionnaire was completed carefully. |  |  |  |  |  |
| Ensuring the patient adhered to follow-up appointments. |  |  |  |  |  |
| Delivering the sample for genetic analysis. |  |  |  |  |  |
| Delivering the electronic bracelet. |  |  |  |  |  |
| Explaining how the electronic bracelet works. |  |  |  |  |  |
| Explaining the results of the analyses (Polygenic Risk Score). |  |  |  |  |  |

The following questions pertain to your **knowledge and attitudes toward polygenic risk scores**.

**Experience with Polygenic Risk Tests**

1. How informed do you feel about polygenic risk tests?
   - Not at all
   - A little
   - Moderately
   - Very
2. Have you ever discussed polygenic risk tests with a patient (including the possibility of such tests becoming available in the future)?
   - No
   - Yes
     If yes, please specify for which condition.
3. Have you ever prescribed polygenic risk tests to assess a patient’s disease risk (select all that apply)?
   - No
   - Yes, for clinical purposes
   - Yes, for research purposes
     If yes, specify for which condition.
4. Have you ever returned polygenic risk test results to a patient (select all that apply)?
   - No
   - Yes, in a clinical setting
   - Yes, as part of a research project
     If yes, specify for which condition.
5. Indicate how confident you would feel in each of the following situations:

| **Situation** | **Not at all** | **A little** | **Moderately** | **Very** |
| --- | --- | --- | --- | --- |
| Discussing polygenic risk tests for disease with the patient. | ❑ | ❑ | ❑ | ❑ |
| Recommending a polygenic risk test to a patient with a family history of disease. | ❑ | ❑ | ❑ | ❑ |
| Answering patient questions about polygenic risk tests. | ❑ | ❑ | ❑ | ❑ |
| Interpreting polygenic risk test results. | ❑ | ❑ | ❑ | ❑ |
| Explaining lifetime disease risk to a patient based on polygenic risk test results. | ❑ | ❑ | ❑ | ❑ |
| Managing a patient with an increased polygenic risk of disease. | ❑ | ❑ | ❑ | ❑ |
| Managing a patient with a reduced polygenic risk of disease. | ❑ | ❑ | ❑ | ❑ |

**Knowledge of Polygenic Risk Scores**

1. For each of the following statements, select **False**, **True**, or **Don’t know**.

| **Statement** | **False** | **True** | **Don’t know** |
| --- | --- | --- | --- |
| Polygenic risk is a cumulative measure of multiple single nucleotide polymorphisms (SNPs). | ❑ | ❑ | ❑ |
| Polygenic risk test results are usually expressed as a risk score. | ❑ | ❑ | ❑ |
| Each individual SNP has a different minor allele frequency, but polygenic risk scores can have a normal distribution in the population. | ❑ | ❑ | ❑ |
| Most SNPs associated with cancer risk have been identified in exons. | ❑ | ❑ | ❑ |
| Genetic recombination is not random, and some variants are often inherited together. | ❑ | ❑ | ❑ |
| Inheritance patterns for SNPs can be explained using pedigrees similar to those used for single-gene variants. | ❑ | ❑ | ❑ |
| Including more SNPs in a polygenic risk calculation means better risk prediction. | ❑ | ❑ | ❑ |
| Polygenic risk is equally accurate across all ethnicities. | ❑ | ❑ | ❑ |
| Polygenic risk is equally accurate for all types of diseases. | ❑ | ❑ | ❑ |
| Incorporating a polygenic risk score into risk prediction models can provide greater precision in predicting the risk of certain cancers. | ❑ | ❑ | ❑ |

**Attitudes Toward Polygenic Risk Tests**

1. Indicate how concerned you are about the following potential limitations of polygenic risk tests:

| **Limitation** | **Uncertain** | **Not at all concerned** | **A little concerned** | **Very concerned** |
| --- | --- | --- | --- | --- |
| The impact of genealogy on interpreting polygenic risk test results. | ❑ | ❑ | ❑ | ❑ |
| The impact of non-genetic factors (e.g., lifestyle and other traditional risk factors) on interpreting polygenic risk test results. | ❑ | ❑ | ❑ | ❑ |
| The possibility that polygenic risk estimates change with age (e.g., due to epigenetic factors). | ❑ | ❑ | ❑ | ❑ |
| The impact of SNPs yet to be identified on current polygenic risk assessments. | ❑ | ❑ | ❑ | ❑ |
| The ability of polygenic risk tests to predict different types of diseases. | ❑ | ❑ | ❑ | ❑ |
| Adverse psychological reactions in patients receiving information about their polygenic risk. | ❑ | ❑ | ❑ | ❑ |
| Negative impacts on the lives of patients receiving information about their polygenic risk. | ❑ | ❑ | ❑ | ❑ |

1. In your opinion, what is the likelihood of the following potential negative consequences of polygenic risk tests?

| **Consequence** | **Very low/none** | **Low** | **Moderate** | **High** | **Very high** |
| --- | --- | --- | --- | --- | --- |
| Psychological distress. | ❑ | ❑ | ❑ | ❑ | ❑ |
| Family conflicts. | ❑ | ❑ | ❑ | ❑ | ❑ |
| Unnecessary screening and treatments. | ❑ | ❑ | ❑ | ❑ | ❑ |
| Increased uncertainty about individual risk. | ❑ | ❑ | ❑ | ❑ | ❑ |
| Insufficient healthcare resources. | ❑ | ❑ | ❑ | ❑ | ❑ |

1. In your opinion, what is the likelihood of potential misuse/abuse of polygenic risk test results by third parties regarding...?

| **Scenario** | **Very low/none** | **Low** | **Moderate** | **High** | **Very high** |
| --- | --- | --- | --- | --- | --- |
| Limited job opportunities. | ❑ | ❑ | ❑ | ❑ | ❑ |
| Barriers to obtaining health insurance coverage. | ❑ | ❑ | ❑ | ❑ | ❑ |
| Stigmatization of the individual. | ❑ | ❑ | ❑ | ❑ | ❑ |

1. Describe any other concerns you have regarding polygenic risk tests:
   ……………………………………………………………………………………………………………………
2. In your opinion, what are the potential benefits of offering polygenic risk tests to patients?

| **Benefit** | **Not at all beneficial** | **Slightly beneficial** | **Somewhat beneficial** | **Beneficial** | **Very beneficial** |
| --- | --- | --- | --- | --- | --- |
| Reducing uncertainty about genetic risk. | ❑ | ❑ | ❑ | ❑ | ❑ |
| Providing information to children. | ❑ | ❑ | ❑ | ❑ | ❑ |
| Providing information to other family members. | ❑ | ❑ | ❑ | ❑ | ❑ |
| Providing information for family planning. | ❑ | ❑ | ❑ | ❑ | ❑ |
| Access to personalized monitoring, screening, or treatment pathways. | ❑ | ❑ | ❑ | ❑ | ❑ |
| Encouraging prevention, including lifestyle changes. | ❑ | ❑ | ❑ | ❑ | ❑ |

1. Describe any other benefits you believe may arise from polygenic risk tests:
   ……………………………………………………………………………………………………………………
2. Considering current knowledge, resources, and technology, indicate to what extent you agree or disagree with the following statements:

| **Statement** | **Strongly disagree** | **Disagree** | **Neutral** | **Agree** | **Strongly agree** |
| --- | --- | --- | --- | --- | --- |
| The expected health benefits of using polygenic risk tests outweigh the negative consequences, justifying their use. | ❑ | ❑ | ❑ | ❑ | ❑ |
| Polygenic risk tests have a role in the context of familial diseases. | ❑ | ❑ | ❑ | ❑ | ❑ |
| Polygenic risk tests should only be offered to individuals at risk of hereditary diseases. | ❑ | ❑ | ❑ | ❑ | ❑ |
| Polygenic risk tests should be offered to the general population to guide prevention and screening programs. | ❑ | ❑ | ❑ | ❑ | ❑ |

1. Are there other scenarios for which you would or would not recommend a polygenic risk test? If yes, please specify:
   ……………………………………………………………………………………………………………………

**Preparation, Requirements, and Propensity**

1. In your opinion, how likely is it that polygenic risk tests will influence patient care in the future?

- Very likely
- Likely
- Uncertain
- Unlikely
- Very unlikely

1. If polygenic risk tests were implemented at the population level to guide prevention and screening programs, what role do you think genetic professionals (genetic counselors, clinical geneticists) should have (select all that apply)?

- Minimal or none
- Supporting specialist physicians who will have a primary role
- Supporting general practitioners (family doctors) who will have a primary role
- Primary
- Other: ……………………………………………………

1. How prepared do you feel for the potential integration of polygenic risk tests into your clinical practice?

- Not at all prepared
- A little prepared
- Adequately prepared
- Very prepared
- Don’t know

1. What resources would you find useful to feel prepared for the practical implementation of polygenic risk tests (select all that apply)?

- Telephone support service for healthcare providers
- Online service at the point of care about polygenic tests (text-based)
- Online service at the point of care about polygenic tests (video-based)
- Patient information sheets about polygenic tests and their implications
- Other (please specify): ……………………………………………………

**Additional Comments (Optional):**
……………………………………………………………………………………………………………………
